# The safety and use of perioperative dexamethasone in the perioperative management of primary sporadic supratentorial meningiomas

**DOI:** 10.3389/fonc.2024.1379692

**Published:** 2024-04-23

**Authors:** Felix Arlt, Alim Emre Basaran, Markus Vogel, Martin Vychopen, Clemens Seidel, Alonso Barrantes-Freer, Erdem Güresir, Johannes Wach

**Affiliations:** ^1^ Department of Neurosurgery, University of Leipzig Medical Center, Leipzig, Germany; ^2^ Comprehensive Cancer Center Central Germany, Partner Site Leipzig, Leipzig, Germany; ^3^ Department of Radiation Oncology, University of Leipzig Medical Center, Leipzig, Germany; ^4^ Paul Flechsig Institute of Neuropathology, University of Leipzig Medical Center, Leipzig, Germany

**Keywords:** dexamethasone, edema, functional outcome, MIB-1, supratentorial meningioma

## Abstract

**Objective:**

Despite the lack of prospective evidence for the perioperative use of dexamethasone in meningioma surgery, its use is well established in the daily routine of several centers. The present study evaluates the effect of dexamethasone on postoperative complications, peritumoral T2/FLAIR hyperintensity, and progression-free survival in patients with supratentorial meningiomas undergoing resection.

**Methods:**

A total of 148 patients who underwent resection of a primary sporadic supratentorial meningioma at the authors’ institution between 2018 and 2020 were included in this retrospective cohort. Safety criteria were side effects of dexamethasone (e.g. hyperglycemia), surgical morbidities, length of stay, and mortality. The individual Karnofsky Performance Scales (KPS) were evaluated regarding the individual development and the delta of KPS at 3- and 12-months compared to baseline KPS was calculated. Longitudinal assessment of the peritumoral T2-/FLAIR hyperintensity changes was performed.

**Results:**

The use of both pre- and postoperative dexamethasone did not influence the incidence rates of wound infections, infarctions, postoperative seizures, pulmonary embolism, postoperative hemorrhage, mortality, length of stay, new-onset hyperglycemia and new neurological deficits. Perioperative Dexamethasone use was associated with an improved Karnofsky performance development at 3- (delta of KPS 3.3 *vs*. -1.9, p=0.001) and 12-months (delta of KPS 3.8 *vs*. -1.1, p=0.008) compared to the preoperative Karnofsky performance status. Multivariable analysis revealed that perioperative dexamethasone use enhances the KPS improvement (OR: 3.65, 95% CI: 1.01-13.18, *p*=0.048). Persistent peritumoral T2/FLAIR hyperintensity changes were observed in 35 cases of 70 patients with available follow-up images and a baseline edema (50.0%). Perioperative dexamethasone use enhanced the reduction of the preoperative peritumoral T2-/FLAIR hyperintensity changes (mean reduction of maximum diameter: 1.8 cm *vs*. 1.1 cm, *p*=0.023). Perioperative dexamethasone use was independently associated with a lower risk for persistent peritumoral T2-/FLAIR hyperintensity changes (OR: 3.77, 95% CI: 1.05-13.54, *p*=0.042) The perioperative use of dexamethasone did not influence the progression-free survival time in Simpson grade I or II resected WHO grade 1 meningiomas (log-rank test: *p*=0.27).

**Conclusion:**

Perioperative dexamethasone use seems to be safe in surgery for primary supratentorial meningiomas. Dexamethasone use might enhance the functionality by reducing postoperative peritumoral T2-/FLAIR hyperintensities. These findings highlight the need for prospective data.

## 1 Introduction

Meningiomas arise from arachnoid cap cells and are the most common type of intracranial tumor among adults (1). Although the majority of meningiomas are classified as World Health Organization grade 1 tumors, approximately 20-30% are WHO grade 2 or 3 tumors with aggressive nature and show meningioma progression despite undergoing complete resection and adjuvant therapy (2). The generation of edema by these tumors can lead to substantial morbidity by exerting pressure on adjacent structures. Approximately 38-67% of individuals diagnosed with intracranial meningiomas suffer from peritumoral brain edema, which might result in increased intracranial pressure and an increased probability of symptom development such as seizures (3–7). Peritumoral brain edema is suggested to be associated with increased proliferative activity, pial blood supply and loss of arachnoid dissection plane (7, 8).

Dexamethasone (Dex) has historically been established for the therapy of brain edema in brain tumor patients (9, 10). The major evidence for the perioperative use of dexamethasone is given in the field of glioma and it has been integrated in some guidelines despite the major studies have been performed in the 1990s (11, 12). However, there is also controversy whether corticosteroid therapy can induce a decrease of peritumoral edema in meningioma (13–17). Due to the lack of specific guidelines for dexamethasone use in the perioperative period, there is high variability regarding Dex application among several centers.

Against this backdrop, the present investigation analyses perioperative Dex-associated side effects, the impact on peritumoral T2-/FLAIR hyperintensities, functionality graded by the Karnofsky Performance status (KPS), and progression-free survival (PFS).

## 2 Methods

### 2.1 Study design and inclusion criteria

This study retrospectively reviews consecutively treated patients with primary sporadic supratentorial meningioma who had undergone surgical resection between 2018 and 2020. The present study adhered to the guidelines outlined in the Declaration of Helsinki and received approval from the local Ethics Committee at the Medical Faculty of the University of Leipzig (No. 117/23-ek). Informed consent for scientific use of anonymized data was signed by all patients.

The following cranial locations were included in the present study: Convexity, falcine, parasagittal, lateral/medial sphenoid wing, olfactory groove, planum ethmoidale-sphenoidale, parasellar, and tuberculum sellae. Patients with cranial infratentorial, intraventricular, and spinal meningiomas, recurrent meningiomas after radiotherapy, patients < 18 years, and patients with a neurofibromatosis type II were excluded due to the differences regarding clinical signs, histopathology, and therapy (18–22). Patients who were lost to follow-up within the initial 4 weeks post-surgery were not included in the analysis. A total of 148 patients fulfilled the inclusion criteria and were eligible for the data analysis. The clinical decision-making process involved an interdisciplinary neuro-oncological board comprised of senior experts in neuro-oncology, neuroradiology, radiotherapy, and neurosurgery. Post-surgery, routine follow-up includes the initial imaging at 3 months and subsequent scans annually for WHO grade 1 meningiomas, while for WHO grade 2 meningiomas, imaging is performed every 6 months (23).

### 2.2 Data recording

**Figure 1:**
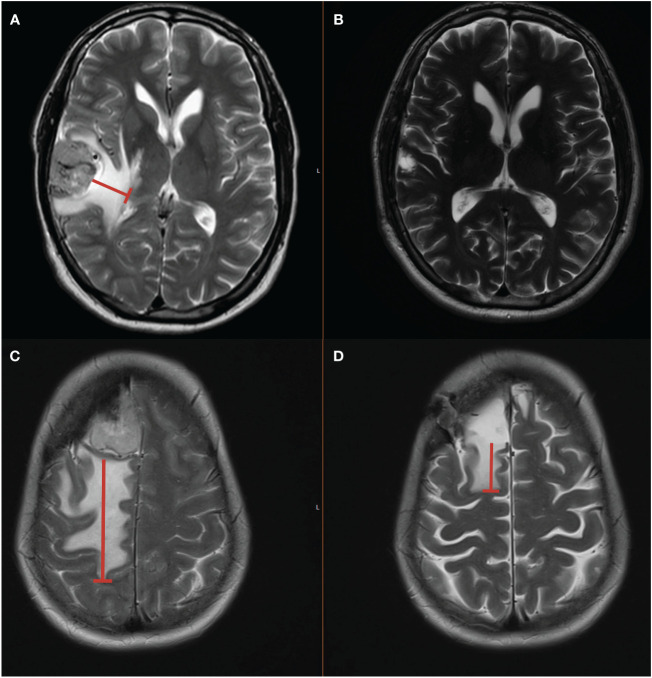
**(A)** T2-weighted MR-image illustrating a right-sided convexity meningioma with preoperative peritumoral edema. **(B)** Shows the 12-month postoperative MRI after Simpson grade I resection and perioperative Dex intake without evidence of residual peritumoral T2-hyperintensity zone. **(C)** Shows a right frontal convexity meningioma with large peritumoral edema in the frontal lobe (red line). **(D)** The patient also underwent a Simpson grade I resection but without Dex intake and peritumoral T2 hyperintensity persists until 36 months after surgery.

To assess the patient characteristics, we analyzed the demographic data, laboratory tests on admission, comorbidities, preoperative neurological status, preoperative WHO classification based on neuropathological assessment, length of stay, new postoperative neurological deficits, postoperative infarction or rebleeding, extent of meningioma resection based on the Simpson classification system in line with the European Association of Neuro-Oncology (EANO) (Simpson grade I-III = gross total resection, Simpson grade IV = subtotal resection, and Simpson grade V = biopsy), in-hospital mortality, and postoperative follow-up data were recorded and entered into a computerized dataset (SPSS, Version 29 for Windows, IBM Corp., Armonk, NY, USA) (23). Preoperative magnetic resonance imaging (MRI) was consistently conducted using high-field strength (3.0 Tesla) for both initial T1-weighted (without and with gadolinium enhancement) and T2-weighted scans, carried out within a 48-hour timeframe before surgery. To ascertain the volume and the tumor surface area, Gadolinium-enhanced T1-weighted MR images were employed. The calculations were carried out through volumetric analysis using 3D Slicer software (version 5.2.1, Surgical Planning Laboratory, Harvard University, USA). The measurement of peritumoral T2-/FLAIR hyperintensities suggested to be peritumoral edema involved determining the maximum reach of increased T2/FLAIR signal intensity along the tumor periphery in the MRI (24). These measurements were performed for the preoperative and the available postoperative MRI-scans (see **Figure 1**). The histological findings were reviewed regarding WHO grade, Molecular Immunology Borstel (MIB)-1 index and the number of mitotic figures. Finally, we reviewed the outpatient reports to assess the postoperative outcomes at 3- and 12-months postoperatively.

### 2.3 Surgical management and clinical outcome parameters

Perioperative institutional Dex schedule included a therapy of 12 mg/d in three single doses. Tapering to reduce Dex was performed if Dex was prescribed for >5 days. The perioperative decision-making process to prescribe, interrupt and stop Dex therapy is summarized in the **Supplementary Figure 1**. All microsurgical resections were performed under general anesthesia using white-light resection guided by neuronavigation (Brainlab Curve, BrainLAB AG, Feldkirchen, Germany) and an operating microscope (Pentero, Carl Zeiss, Jena, Germany). Postoperative complications occurring immediately after surgery were defined as the presence of venous thrombosis, infarction, seizures, rebleeding, new motor or sensory deficits, new brain nerve deficits between the date of tumor resection and discharge from the neurosurgical ward. Postoperative wound infections were defined as surgical site infections between the date of tumor resection and the date of the follow-up appointment at 3-month at our outpatient clinic. Hyperglycemia that develops for the first time was characterized as clinically documented elevated blood sugar occurring between the surgical date and the 3-month clinic follow-up assessment in a patient without a pre-existing diagnosis of diabetes mellitus. Progression of meningioma was defined as the emergence of new local lesions or the expansion of progressively remaining meningioma at the location of the initial surgery during a subsequent MRI examination (25). PFS time was defined as the timeframe between the date of surgical resection and the date of tumor progression or last known follow-up appointment.

### 2.4 Immunohistochemistry

Hematoxylin and eosin (H&E) were used to stain paraffin sections. Immunohistochemical analysis was conducted on the specimen, using the Molecular Immunology Borstel-I (MIB-I, DAKO, Glostrup, Denmark) antibody for Ki67 antigen detection. The MIB-I index was determined using the average method as previously described (26).

### 2.5 Statistics

Data were organized and analyzed using SPSS for Windows (version 29.0; IBM Corp., Armonk, NY, USA). Fisher´s exact test (two-sided) was performed to compare nominal variables and the student´s t-test for metric variables between patients with and without perioperative dexamethasone use. Only two-sided p-values are reported. Violin-Plots were created using GraphPad Prism 8 (GraphPad Software, San Diego, CA, USA) to visualize the change of KPS and peritumoral T2-/FLAIR hyperintensities in those with or without perioperative dexamethasone intake. Receiver-operating characteristics (ROC) curve analysis was performed to identify the optimum cut-off value of the MIB-1 index in predicting the course of peritumoral T2-/FLAIR hyperintensities. ROC curve was created using ggplot2 in R. Multivariable binary logistic regression analyses were performed to analyze factors being associated with change in KPS from baseline to 3-months after surgery and development of peritumoral T2-/FLAIR hyperintensities. Sankey plots were created with the online RAWGraphs and modified with BioRender (27). We performed log-rank tests and created Kaplan-Meier charts of PFS in WHO grade 1 meningiomas who underwent Simpson grade I or II resections using the R package *Survminer* and *Survival* in R software version 4.3.1 (R Foundation for Statistical Computing, Vienna, Austria).

## 3 Results

### 3.1 Patient characteristics

Patient characteristic showed that 103 (69.6%) patients were female, and 45 patients (30.4%) were male. The median age was 62.0 (IQR 52.3-72.0). WHO grade 1 meningiomas were found in 139 (69.6%) patients and WHO grade 2 meningiomas in 9 patients (6.1%). The median preoperative KPS was 80.0 (IQR: 80.0-90.0). Fifty-five (55/148; 37.2%) patients reported to be a former or current smoker, and 20 (20/148; 13.5%) had a diabetes in their medical history.

The median tumor surface area was 41.1 cm^2^ (IQR: 19.5-78.5) and in 70 patients (70/148; 47.3%) peritumoral T2-/FLAIR hyperintense edema areas with a median maximum diameter of 2.1 cm was observed. Seventy-six patients (51.4%) did not receive perioperative dexamethasone, 4 (2.7%) received only preoperative dexamethasone, 18 (12.2%) only postoperative dexamethasone, and 50 (33.8%) received both pre- and postoperative dexamethasone. Simpson grade I or II resections were performed in 120 patients (120/148; 81.1%) and grade ≥III resections were performed in 28 patients (18.9%). Concerning immediate postoperative complications, 1.4% (2/148) of individuals experienced venous thrombosis/pulmonary embolism, 14.4% (21/148) had new-onset or worsened seizures, 2.0% (3/148) suffered a stroke, 5.4% (8/148) exhibited a postoperative wound infection, and 10.1% (15/148) developed a worsening of preoperative dysfunctions or new motor, sensory or brain nerve deficits. New-onset hyperglycemia was found in 2.0% of the cohort. In-hospital mortality was observed in one case (0.7%) from pulmonary embolism. The median KPS at 3-months after surgery was 80.0 (IQR: 72.5-90.0). Further details are summarized in **Table 1**.

**Table 1:** Patient characteristics (n = 148).

Median age (IQR) (in y)	62.0 (52.3-72.0)
Sex Female Male	103 (69.6%) 45 (30.4%)
Diabetes mellitus	20 (13.5%)
Former or current smoker	55 (37.2%)
Preoperative Karnofsky Performance Status, median (IQR)	80 (80.0-90.0)
Tumor surface area, cm^2^, median (IQR)	41.1 cm^2^ (19.5-78.5)
Preoperative edema	70 (47.3%)
Peritumoral edema, cm, median (IQR)	2.1 (1.4-3.4)
Calcification	6 (4.1%)
Skull-base Non-Skull base	55 (37.2%) 93 (62.8%)
Dexamethasone use None Preop only Postop only Pre- & postop	76 (51.4%) 4 (2.7%) 18 (12.2%) 50 (33.8%)
Simpson grade Simpson grade I & II Simpson grade ≥III	120 (81.1%) 28 (18.9%)
WHO grade 1 2	139 (93.1%) 9 (6.1%)
MIB-1 index, Median (IQR) (available in 128 patients)	3.0 (2.0-4.0)
Length of stay, days, median (IQR)	7.0 (5.0-8.0)
Immediate postoperative complications Venous thrombosis/pulmonary embolism New-Onset or worsened Seizures Stroke Wound infections Worsening or new motor, sensory, or brain nerve deficits	2 (1.4%) 21 (14.4%) 3 (2.0%) 8 (5.4%) 15 (10.1%)
New-onset hyperglycemia	3 (2.0%)
In-hospital mortality	1 (0.7%)
Karnofsky Performance Status at 3-months, median (IQR)	80.0 (72.5-90.0)
Karnofsky Performance Status at 12-months, median (IQR)	90.0 (80.0-90.0)

IQR, Interquartile range; KPS, Karnofsky Performance Status; MIB-1, Molecular Immunology Borstel-1; WHO, World Health Organization; Y, years.

### 3.2 Univariable analysis

The analysis of baseline patient characteristics was conducted through univariable analysis, with stratification based on those with or without perioperative dexamethasone use (see **Table 2**). There was a tendency towards perioperative Dex use in older patients (63.2 ± 12.9 *vs* 59.4 ± 12.6, *p* = 0.07). Furthermore, an observable trend towards perioperative Dex administration was noted in those with a larger peritumoral edema (2.7 ± 1.8 *vs*. 2.0 ± 1.5, *p* = 0.08). A notable discrepancy towards a lower preoperative KPS (76.1 ± 11.3; *p* < 0.001) and a higher tumor surface area (70.7 cm^2^ ± 39.9 *vs*. 39.7 ± 52.4; *p* < 0.001) among those with perioperative Dex use was observed. The distributions of sex, diabetes mellitus, smoking status, calcification, extent of resection, WHO grade, and MIB-1 index did not differ between those with or without perioperative Dex use.

**Table 2:** Comparison of patient characteristics in groups with perioperative dexamethasone use and without perioperative dexamethasone (using Fisher’s exact test (two-sided) and independent *t*-test).

Characteristics	Dexamethasone use (72/148; 48.6%)	Nodexamethasone use (76/148; 51.4%)	*p*-value
Age (years), mean ± SD	63.2 ± 12.9	59.4 ± 12.6	0.07
Sex Female Male	46 (63.9%) 26 (36.1%)	57 (75.0%) 19 (25.0%)	0.16
Diabetes mellitus	13 (18.1%)	7 (9.2%)	0.15
Former or current smoker	30 (41.7%)	25 (32.9%)	0.31
Preoperative KPS, mean ± SD	76.1 ± 11.3	82.8 ± 8.3	*< 0.001*
Tumor surface area, cm^2,^ mean ± SD	70.7 ± 39.9	39.7 ± 52.4	*< 0.001*
Peritumoral edema, cm, mean ± SD	2.7 ± 1.8	2.0 ± 1.5	0.08
Calcification	2 (2.8%)	4 (5.3%)	0.68
Simpson grade ≤II >II	60 (83.3%) 12 (16.7%)	60 (78.9%) 16 (21.1%)	0.54
WHO grade 1 2	66 (91.7%) 6 (8.3%)	73 (96.1%) 3 (3.9%)	0.32
MIB-1 index, mean ± SD	3.7 ± 2.1	3.7 ± 2.7	0.98

*P*-values in italic represent statistically significant results.

KPS, Karnofsky Performance Status; MIB-1, Molecular Immunology Borstel-1; SD, Standard deviation; WHO, World Health Organization.

### 3.3 Immediate postoperative patient outcomes stratified by perioperative dexamethasone use

We investigated the impact of perioperative Dex administration on postoperative patient outcomes, as detailed in **Table 3**. Concerning immediate postoperative complications, there were no statistically significant differences in the occurrence of venous thrombosis/pulmonary embolism (1.4% *vs*. 1.3%, *p* = 0.99), worsened or new-onset seizures (15.3% *vs*. 13.2%, *p* = 0.82), stroke (2.8% *vs*. 1.3%, *p* = 0.61), wound infections (4.2% *vs*. 6.6%, *p* = 0.72), or new or worsened motor, sensory, or brain nerve deficits (12.5% *vs*. 7.9%, *p* = 0.42). There was no significant difference in in-hospital mortality between patients with perioperative Dex use and those without (1.4% *vs*. 0.0%, *p* = 0.11). New-onset hyperglycemia was observed in three patients with perioperative Dex use, whereas no new-onset hyperglycemia was found in those without receiving Dex perioperatively (*p* = 0.11). The mean length of stay was slightly higher in the perioperative Dex group compared to the non-Dex group, but the difference was not statistically significant (8.6 ± 3.9 days *vs*. 7.2 ± 4.7 days, *p* = 0.06).

**Table 3:** Comparison of patient outcomes in groups with perioperative dexamethasone use and without perioperative dexamethasone (using Fisher’s exact test (two-sided) and independent *t*-test).

Outcome	Dexamethasone use (72/148; 48.6%)	No dexamethasone use (76/148; 51.4%)	*p*-value
Immediate postoperative complications Venous thrombosis/pulmonary embolism New-Onset Seizures Stroke Wound infections Worsening or new motor, sensory, or brain nerve deficits	1 (1.4%) 11 (15.3%) 2 (2.8%) 3 (4.2%) 9 (12.5%)	1 (1.3%) 10 (13.2%) 1 (1.3%) 5 (6.6%) 6 (7.9%)	0.99 0.82 0.61 0.72 0.42
In-hospital mortality	1 (1.4%)	0 (0.0%)	0.49
New-onset hyperglycemia	3 (4.2%)	0 (0.0%)	0.11
Length of stay, days, mean ± SD	8.6 ± 3.9	7.2 ± 4.7	0.06
Reduction of extent of peritumoral T2-/FLAIR Hyperintensity (baseline *vs*. last follow-up MRI), cm, mean ± SD	1.8 ± 1.5	1.1 ± 0.9	*0.04*
Change in KPS, baseline - 3 months, mean ± SD	3.8 ± 9.8	-2.1 ± 9.9	*0.001*
Change in KPS, baseline - 12 months, mean ± SD	4.3 ± 8.3	-1.1 ± 9.6	*0.004*

*P*-values in italic represent statistically significant results.

KPS, Karnofsky Performance Status; SD, Standard deviation.

### 3.4 Impact of perioperative dexamethasone use on peritumoral T2-/FLAIR hyperintensities

**Figure 2:**
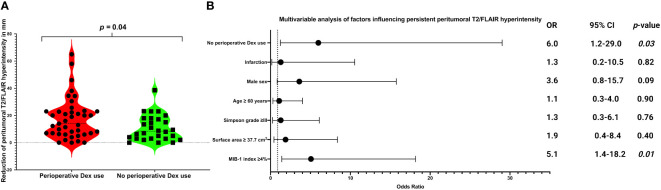
**(A)** Violin-plots displaying reduction of peritumoral T2/FLAIR hyperintensity (in mm) from baseline to last follow-up image in in patients with perioperative Dex intake (red) and without perioperative Dex intake (green). Points and squares indicate the individual values. Median values are presented by the dotted black lines (*p*-values of the Student´s *t*-test). **(B)** Forest plots from multivariable binary logistic regression analysis: Perioperative Dex intake and low MIB-1 labeling index (<4%) was found to be associated with a lower probability of persistent peritumoral T2/FLAIR hyperintensity.

Persistent peritumoral T2-/FLAIR hyperintensities were observed in 35 cases of 70 with a preoperative peritumoral edema (35/70; 50.0%). A statistically significant difference was observed in the reduction of peritumoral T2-/FLAIR hyperintensity between baseline and last follow-up MRI, with a higher mean reduction in the perioperative Dex group compared to the non-Dex group (1.8 ± 1.5 cm *vs*. 1.1 ± 0.9 cm, *p* = 0.04). **Figure 2A** displays violin plots that summarize and compare the distribution of peritumoral T2-/FLAIR hyperintensity change between those who received perioperative Dex or not. MIB-1 index was available in 68 of those 70 cases with a preoperative tumor edema. The optimum cut-off of MIB-1 index in the prediction of persisting T2/FLAIR abnormalities has been determined using a Receiver-operating characteristic curve analysis. The area under curve for MIB-1 index in identifying those with persistent T2-/FLAIR abnormalities was 0.67 (95% CI: 0.53-0.80, *p* = 0.019). The optimum cut-off has been identified at ≥4/<4% with a sensitivity and specificity, respectively (see **Supplementary Figure 2**). Twenty-three (23/34; 67.6%) patients of those with an MIB-1 index ≥4% had persistent peritumoral T2-/FLAIR hyperintensities at the last follow-up examination, whereas 11 (11/34; 32.4%) of those with an MIB-1 index <4% had persistent peritumoral T2-/FLAIR hyperintensities (Fisher´s exact test (two-sided): *p* = 0.007). Persistent peritumoral edema did not influence the postoperative onset of new seizures or worsened frequency of seizures. Among those 35 cases with a persistent peritumoral edema 5 patients (5/35; 14.3%) had a postoperative deterioration, whereas two patients (2/35; 5.7%) of those 35 cases with a no longer detectable peritumoral edema in the follow-up had an immediate postoperative worsening or new-onset of seizures (Fisher’s exact test (two-sided: *p* = 0.43). Thirteen (13/20; 65.0%) male patients had persistent peritumoral T2-/FLAIR hyperintensities, and 22 (22/50; 44.0%) female patients had persistent peritumoral T2-/FLAIR hyperintensities, respectively (Fisher´s exact test (two-sided): *p* = 0.185). 52.6% (30/57) underwent a Simpson grade I or II resection and had persistent peritumoral T2-/FLAIR hyperintensities, and 38.5% (5/13) of those who underwent a Simpson grade ≥III resection had persistent peritumoral T2-/FLAIR hyperintensities (*p* = 0.54). Multivariable binary logistic regression analysis considering the following variables has been performed: Perioperative Dex use (with/without), Simpson grade (≤II/>II), tumor surface area (<37.7 cm^2^/≥37.7 cm^2^), age (<60/≥60 years), sex (female/male), infarction (yes/no), and MIB-1 index (<4%/≥4%). The multivariable analysis revealed that MIB-1 index ≥4% (OR: 5.1, 95% CI: 1.4-18.2, *p* = 0.01) and the absence of perioperative Dex intake (OR: 6.0, 95% CI: 1.2-29.0, *p* = 0.03) are independently associated with persistent peritumoral T2/FLAIR hyperintensities. **Figure 2B** shows forest plots summarizing the findings of the multivariable binary logistic regression analysis.

### 3.5 Impact of perioperative dexamethasone use on course of Karnofsky performance scale

**Figure 3:**
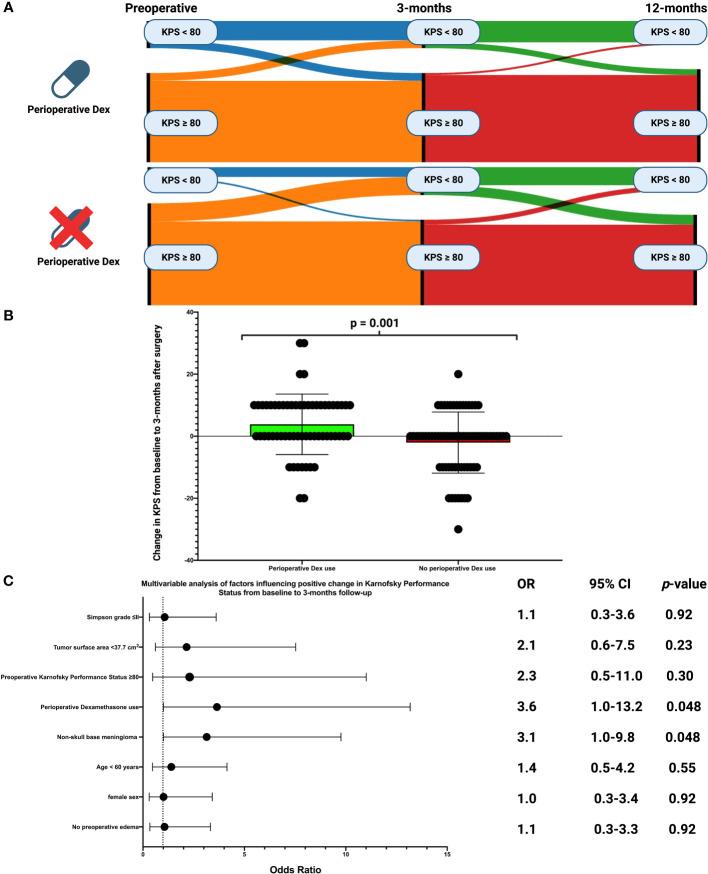
**(A)** Sankey plots of the course of Karnofsky Performance Status scale (dichotomized into poor (<80 = blue/≥80 = orange) of patients with and without perioperative Dex intake from baseline to 12-months after surgery. **(B)** Box-plots displaying the change of the Karnofsky Performance Status scale from baseline to 3-months after surgery in patients with perioperative Dex intake (green) and those without Dex intake (red). The circles indicate the individual values. P-Value of the Student´s *t*-test is given. **(C)** Forest plots from multivariable binary logistic regression analysis: Perioperative Dex intake and non-skull base meningioma was found to be associated with a higher probability of Karnofsky Performance Status improvement from baseline to 3-months after surgery.

The course of the KPS dichotomized into good (KPS ≥80) and low (KPS <80) from baseline over 3-months to 12-months after surgery is shown in Sankey diagrams for those with or without perioperative Dex (see **Figure 3A**). KPS data at 3-months after surgery were available in 128 patients (128/148; 86.5%). Clinical deterioration of the KPS at 3-months was observed in 30 patients (30/128; 23.4%). Deterioration of the KPS at 3-months was observed in 9 cases among those receiving perioperative Dex (9/60; 15.0%), and in 21 among those not receiving perioperative Dex (21/68; 30.9%), respectively (Fisher´s exact test (two-sided): *p* = 0.039). Furthermore, significant differences were found in the change in KPS between baseline and 3-months (3.8 ± 9.8 *vs*. -2.1 ± 9.9, *p* = 0.001, see **Figure 3B**) after surgery as well as between baseline and 12-months (4.3 ± 8.3 *vs*. -1.1 ± 9.6, *p* = 0.004) after surgery. Patients who underwent surgery for a supratentorial skull base meningioma had a mean KPS change from baseline to 3-months after surgery of -1.0 +/- 8.5 compared to 1.8 +/- 11.1 in those who underwent surgery for a supratentorial non-skull base meningioma (independent *t*-test: *p* = 0.13). Elderly patients (≥60 years) showed a delta of the KPS at 3-months compared to baseline of 1.6 +/- 9.8, whereas those under 60 years had a mean KPS change of -0.3 +/- 10.7 (independent *t*-test: *p* = 0.29). Patients who underwent Simpson grade ≤II resection had a mean KPS change from baseline to 3-months after surgery of 1.1 +/- 10.0 compared to -0.8 +/- 11.2 in those who underwent a Simpson grade >2 resection (independent *t*-test: *p* = 0.41).A multivariable binary logistic regression analysis was carried out, considering the following listed variables: Simpson grade (≤II/>II), tumor surface area (<37.7 cm^2^/≥37.7 cm^2^), preoperative KPS (<80/≥80), perioperative Dex use (with/without), location (non-skull base/skull base), age (<60/≥60 years), sex (female/male), and preoperative edema (with/without). The multivariable analysis showed that patients with perioperative Dex intake (OR: 3.6, 95% CI: 1.0-13.2, *p* = 0.048) and those with a non-skull base tumor (OR: 3.1, 95% CI: 1.0-9.8, *p* = 0.048) were independently associated with a lower risk of a clinical deterioration of the KPS from baseline to 3-months after surgery. **Figure 3C** illustrates forest plots summarizing the findings of the multivariable binary logistic regression analysis.

### 3.6 Perioperative dexamethasone therapy and progression-free survival

**Figure 4:**
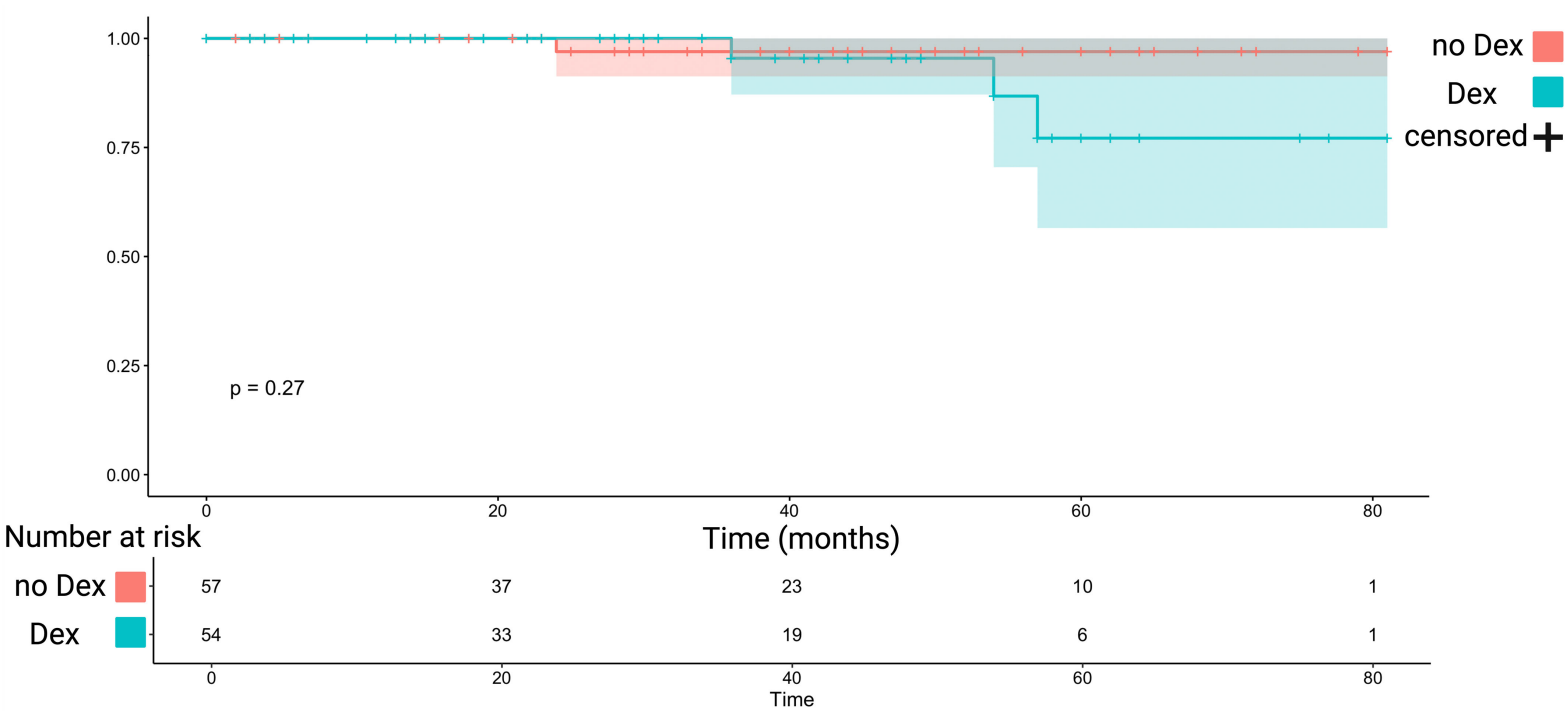
Kaplan-Meier chart displaying probability of progression-free survival stratified by perioperative Dex intake (turquoise) and without perioperative Dex intake (red) in Simpson grade I or II resected WHO grade 1 meningiomas. The shadowed areas represent the 95% confidence intervals. The log-rank test (*p* = 0.27) showed no significant impact of Dex intake on progression-free survival.

PFS analysis was performed in WHO grade 1 tumors who underwent Simpson grade I or II resections. 111 (111/148; 75.0%) fulfilled this inclusion criteria for PFS analysis. The mean follow-up time was 31.7 +/- 22.9 months. Three recurrences were observed in those receiving perioperative Dex (3/54; 5.6%), and 1 patient had a recurrence in those not receiving perioperative Dex (1/57; 1.8%). The mean time to tumor progression in those receiving perioperative Dex therapy was 74.3 months (+/- 3.5), and 79.3 (+/-1.6) months, respectively (log-rank test: *p* = 0.27). **Figure 4** shows the Kaplan-Meier analysis of PFS, which demonstrated no significant differences between those receiving perioperative Dex or not.

## 4 Discussion

The present study investigated the impact of perioperative Dex therapy in sporadic primary supratentorial meningioma surgery regarding Dex-associated side effects, perioperative surgical complications, change of peritumoral T2-/FLAIR hyperintensities, functional outcome, and PFS. Our findings revealed that perioperative Dex intake seems to be safe, facilitates postoperative functionality by enhancing positive KPS change, decreases peritumoral hyperintense T2-/FLAIR lesions, and does not impact tumor progression in WHO grade 1 meningiomas.

The overall survival (OS) and PFS of meningioma patients are influenced by various factors, with the quality of surgery being a significant contributor. Therefore, when examining risk factors related to OS, it is essential to include a discussion on the safety of the surgical procedure. Parameters such as surgical mortality, postoperative hematoma rate, infarction, deep postoperative wound infections, and neurological deterioration post-surgery are widely recognized as reliable indicators of surgical quality. Perioperative Dex intake is widely used and is suggested to facilitate surgery by reducing brain edema and mass effect (28). The present investigation revealed that a majority of individuals undergoing supratentorial meningioma surgery with Dex therapy received Dex therapy both pre- and postoperatively (50/78; 64.1%). The administration of Dex before and after meningioma resection is a common practice due to the prevalent manifestation of cerebral edema in a significant subgroup of these patients. Against this backdrop, Dex therapy was significantly more often prescribed in those with a worse baseline KPS (mean KPS: 76.1 *vs*. 82.8) and larger tumors (mean surface area in cm^2^: 70.7 *vs*. 39.7).

Postoperative infections, thrombosis, and drug associated side effects cause morbidity, prolonged length of hospital stay, and elevated treatment costs (29, 30). Within the purview of this study, patients subjected to perioperative Dex therapy exhibited a non-significant tendency towards an increased occurrence of new-onset hyperglycemia (*p* = 0.11).

For steroid-induced hyperglycemia in meningioma surgery, insulin remains the treatment of choice, especially when glucose exceeds 200 mg/dL (31, 32). Patients with a history of diabetes require tailored insulin adjustments, generally a 20% increase in basal insulin with glucocorticoid initiation (33). In severe hyperglycemia, insulin infusion is indicated. Insulin therapy must be adapted to individual glucose levels, with adjustments based on ongoing glucocorticoid therapy and regular glucose monitoring. Additionally, it is noteworthy that there was not statistically increase in the occurrence of deep venous thrombosis (*p* = 0.99) and wound infections among patients administered perioperative Dex therapy (*p* = 0.72). These results indicate that individuals receiving perioperative Dex therapy might not be at statistically elevated risk for typical complications being associated with Dex, namely wound infection, deep venous thrombosis, and hyperglycemia. This finding is in line with the results of a retrospective study investigating perioperative Dex therapy in 435 glioblastoma patients, which also found no significantly increased risk for these Dex-associated perioperative side effects in glioblastoma patients (34).

Subsequently, we examined whether patients administered perioperative Dex exhibited a higher degree of peritumoral T2-/FLAIR hyperintensity zone reduction. Our findings revealed a noteworthy result, as patients who received perioperative Dex therapy displayed a significantly greater peritumoral T2-/FLAIR hyperintensity zone reduction compared to those who did not receive Dex treatment (1.8 cm *vs*. 1.1 cm, *p* = 0.04). This outcome is also dependent on the clinical rationale for prescribing perioperative Dex in cases with large brain edema. Peritumoral brain edema in meningioma is suggested to be associated with an increased risk of pre- and postoperative seizures as well as postoperative neurological deficits (35, 36). Following intracranial meningioma resection, the disappearance of peritumoral brain edema is occasionally noted in the subsequent months, with the timeframe varying between the individuals (37). Despite prior studies using computer tomography for follow-up reporting persistent edema, Stevens et al. found that 13% of patients experienced postoperative peritumoral brain edema for at least three months (38). However, there are also studies reporting edema to be persistent in up to 30% of cases at a 12-mont follow-up (39). MR images of persistent edema and gliosis on FLAIR and T2 sequences share significant common characteristics, complicating differentiation (40). Hence, persistent edema is suggested to represent a blend of vasogenic brain edema and cerebral gliosis (41). The persistence and prevalence of postoperative T2/FLAIR hyperintensity in those patients with baseline hyperintense findings varied between 39% and 83% at the last follow-up examinations according to a recent systematic review (42). In the present series we observed the presence and persistence of these areas in 50% of the cases with baseline edema and available follow-up images. Hence, we performed a multivariable analysis regarding the persistence of peritumoral T2-/FLAIR hyperintense zones in those with a baseline peritumoral zone suggested to be a brain edema. We identified that meningiomas with an increased MIB-1 labeling index and those who did not receive perioperative Dex therapy were associated with an increased likelihood of persistent postoperative T2/FLAIR hyperintensity zones. MIB-1 labeling index is an established marker for proliferative activity in meningiomas and has been found to be associated with the presence of preoperative brain edema in meningioma patients (8, 43, 44). Hence, the persistence of this area being suggested as a composition of vasogenic brain edema and cerebral gliosis seem to be more frequent among more aggressive meningiomas. Furthermore, dexamethasone is known to have anti-inflammatory effects potentially ameliorating the process of gliosis, which is also potential compound of these persistent T2-/FLAIR hyperintensity zones (45).

Dex was found to have no influence on tumor progression in this series of predominantly WHO grade 1 meningiomas. An important topic for future series will be the role of Dex therapy in WHO grade 3 meningiomas and the management of Dex before and during radiotherapy. corticosteroid use during adjuvant radiation in meningioma requires caution due to its impact on survival and treatment efficacy, as reported in glioma therapies (46). Current practices for adjuvant radiotherapy in WHO grade 3 meningioma, based on EANO guidelines and various studies, are not uniform, with some showing no survival benefit in older patients (47, 48), while others suggest improved outcomes (49). However, it is advised to limit steroid use for asymptomatic or mildly symptomatic edema due do the risks of prolonged administration. Long-term steroid use may also negatively impact survival of WHO grade 3 meningioma due to the known impeding of effectiveness of radiotherapy, chemotherapy, and immunotherapy in glioblastoma (46).

In our follow-up analysis regarding functionality, we observed an association between the perioperative Dex use and an improved KPS change at 3- and 12-months after surgery. Specifically, the most pronounced effect was observed at 3-months (OR: 3.6, 95% CI: 1.0-13.2, *p* = 0.048). This finding was persistent after multivariable analysis with consideration of the baseline KPS, which significantly influences the decision-making process whether Dex is prescribed in the daily clinical practice. Furthermore, we found that that non-skull base meningioma patients had a significantly enhanced KPS course compared to the skull-base meningioma patients (OR: 3.1, 95% CI: 1.0-9.8, *p* = 0.048). This finding is in line with a retrospective study investigating 1148 patients which showed that neurological deterioration is significantly more often observed among skull base meningioma patients (50).

### 4.1 Limitations and future directions

The inherent retrospective nature of this investigation imposes limitations. Manual determination of the KPS score was necessary, as it was not always documented in patient charts. Additionally, the exclusive treatment of the total cohort at a singular institution may compromise the generalizability of the findings. Furthermore, the exact pathophysiological understanding of persistent T2-/FLAIR hyperintensity zones in each individual is not entirely clear which also potentially reduces the generalizability of a beneficial perioperative Dex therapy. However, we applied highly selective inclusion criteria and considered relevant potential confounding variables in our multivariable analyses to overcome these potential issues. Furthermore, the present study represent a monocentric investigation with a limited sample size and further multicentric approaches with large-scale data are necessary to validate these findings. Radiomic features such as shape based on pretherapeutic MR-images have been found to be correlated with WHO grading and MIB-1 labeling index of cranial meningiomas (51, 52). Hence, future studies have to analyze whether artificial neural networks or machine learning based approaches can use pretherapeutic characteristics such as radiomic features to identify those meningioma patients potentially benefiting from corticosteroid therapy.

## 5 Conclusion

The perioperative Dex use appears to be safe during primary supratentorial meningioma surgery. It could potentially improve functionality measured using KPS by diminishing postoperative peritumoral T2-/FLAIR hyperintensity zones. These results underscore the importance of gathering prospective data.

## Data availability statement

The original contributions presented in the study are included in the article/**Supplementary Material**. Further inquiries can be directed to the corresponding author.

## Ethics statement

The studies involving humans were approved by Ethics committee of University of Leipzig. The studies were conducted in accordance with the local legislation and institutional requirements. The ethics committee/institutional review board waived the requirement of written informed consent for participation from the participants or the participants’ legal guardians/next of kin because Written informed consent was waived because the study design has a retrospective nature.

## Author contributions

FA: Conceptualization, Formal analysis, Investigation, Methodology, Writing – original draft. AB: Project administration, Writing – review & editing. MVo: Data curation, Writing – review & editing. MVy: Writing – review & editing. CS: Writing – review & editing. AB-F: Writing – review & editing. EG: Supervision, Resources, Writing – review & editing. JW: Conceptualization, Data curation, Methodology, Software, Supervision, Validation, Visualization, Writing – original draft.
