# Supplementary material for: The safety and use of perioperative dexamethasone in the perioperative management of primary sporadic supratentorial meningiomas
Source: Front Oncol. 2024 Apr 23;14:1379692. doi: 10.3389/fonc.2024.1379692 (PMC11074443; doi:10.3389/fonc.2024.1379692)
Supplement: Supplementary file 1 [file DataSheet_1.pdf]

Preoperative Phase:
Steroids, typically dexamethasone, are often initiated preoperatively to reduce cerebral edema and intracranial pressure in patients with symptomatic brain tumors.
The dose is individualized based on the patient's symptoms, tumor characteristics, and degree of edema.

Intraoperative Phase:
Steroids are continued during surgery to maintain the reduction in cerebral edema and to potentially reduce the risk of perioperative complications.

Immediate Postoperative Phase (day 1-3):
High-dose steroids are usually continued in the immediate postoperative period to manage cerebral edema.
A postoperative MRI within 48 hours can help assess residual tumor and edema, guiding further steroid management.

Postoperative Taper:
The decision to taper steroids should be individualized based on the patient's neurologic status and the extent of cerebral edema.
A common practice is to begin tapering steroids within the first postoperative week, as seen in sources
The tapering schedule can vary, but a gradual reduction over 1 to 2 weeks is typical, starting on the fourth or fifth postoperative day, as mentioned in source

Long-term Management:
Long-term steroid use is associated with significant side effects, including immunosuppression, hyperglycemia, and increased risk of infection.
Therefore, the goal is to minimize the duration of steroid therapy while adequately controlling symptoms.
In cases where steroids cannot be discontinued due to persistent edema or symptomatology, the lowest effective dose should be used, and patients should be monitored for side effects.

Monitoring and Adjustments:
Patients should be monitored closely for signs of steroid-related complications, as well as for recurrence of symptoms that may necessitate adjustments in dosing.
Blood glucose levels should be monitored, and glycemic control should be optimized to reduce the risk of surgical site infections (SSIs), as hyperglycemia can be exacerbated by steroid use.

The newly created supplementary figure 1, which summarizes the institutional Dex treatment strategy for cranial supratentorial meningiomas.

Dexamethasone therapy

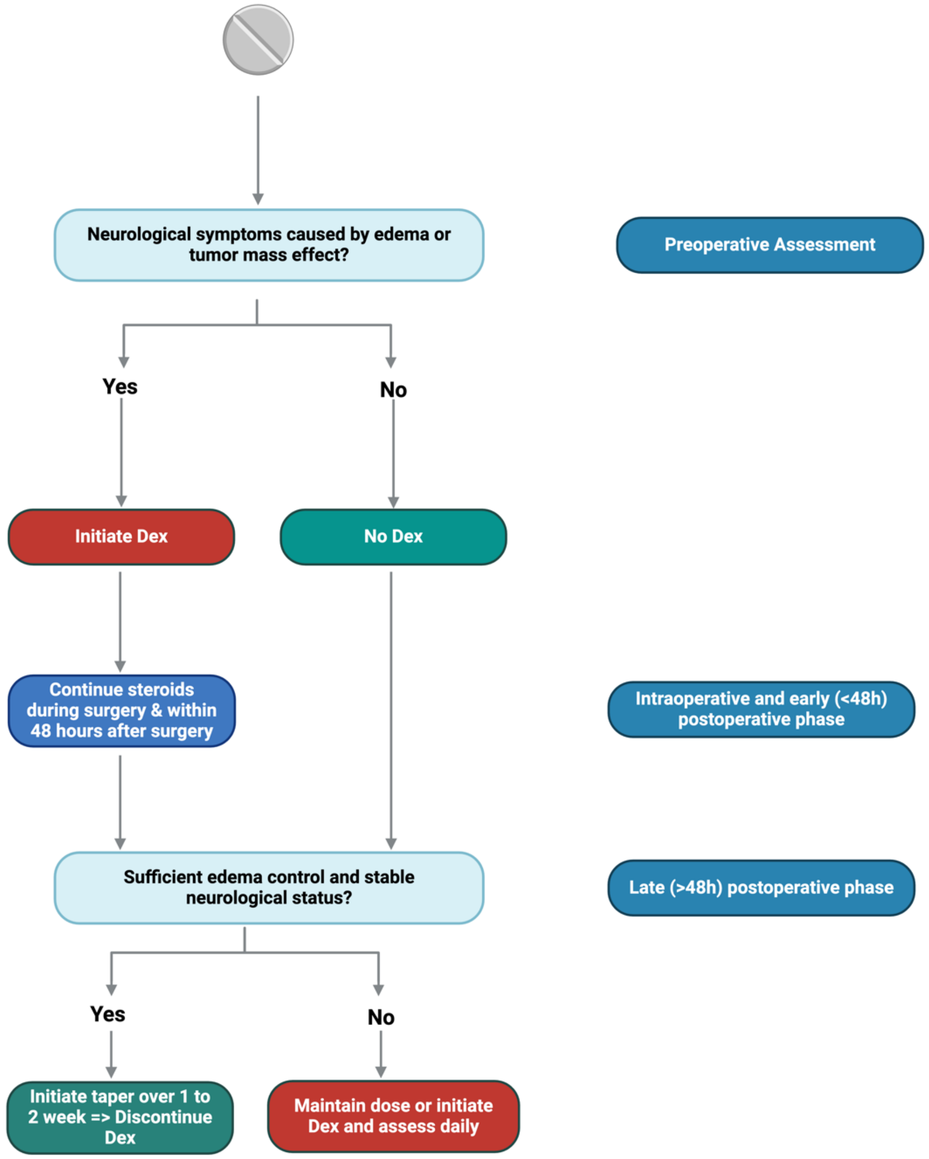

Long-term Management:

Aim to minimize duration of steroid therapy as much as possible

- If steroids can be discontinued, end taper.
- If steroids cannot be discontinued, use the lowest effective dose and monitor for side effects.

Monitoring

Patients should be monitored closely for signs of steroid-related complications

- Blood glucose levels should be monitored, and glycemic control should be optimized to reduce the risk of surgical site infections (SSIs)
