# Supplementary figures and images for: The safety and use of perioperative dexamethasone in the perioperative management of primary sporadic supratentorial meningiomas
Source: Front Oncol. 2024 Apr 23;14:1379692. doi: 10.3389/fonc.2024.1379692 (PMC11074443; doi:10.3389/fonc.2024.1379692)

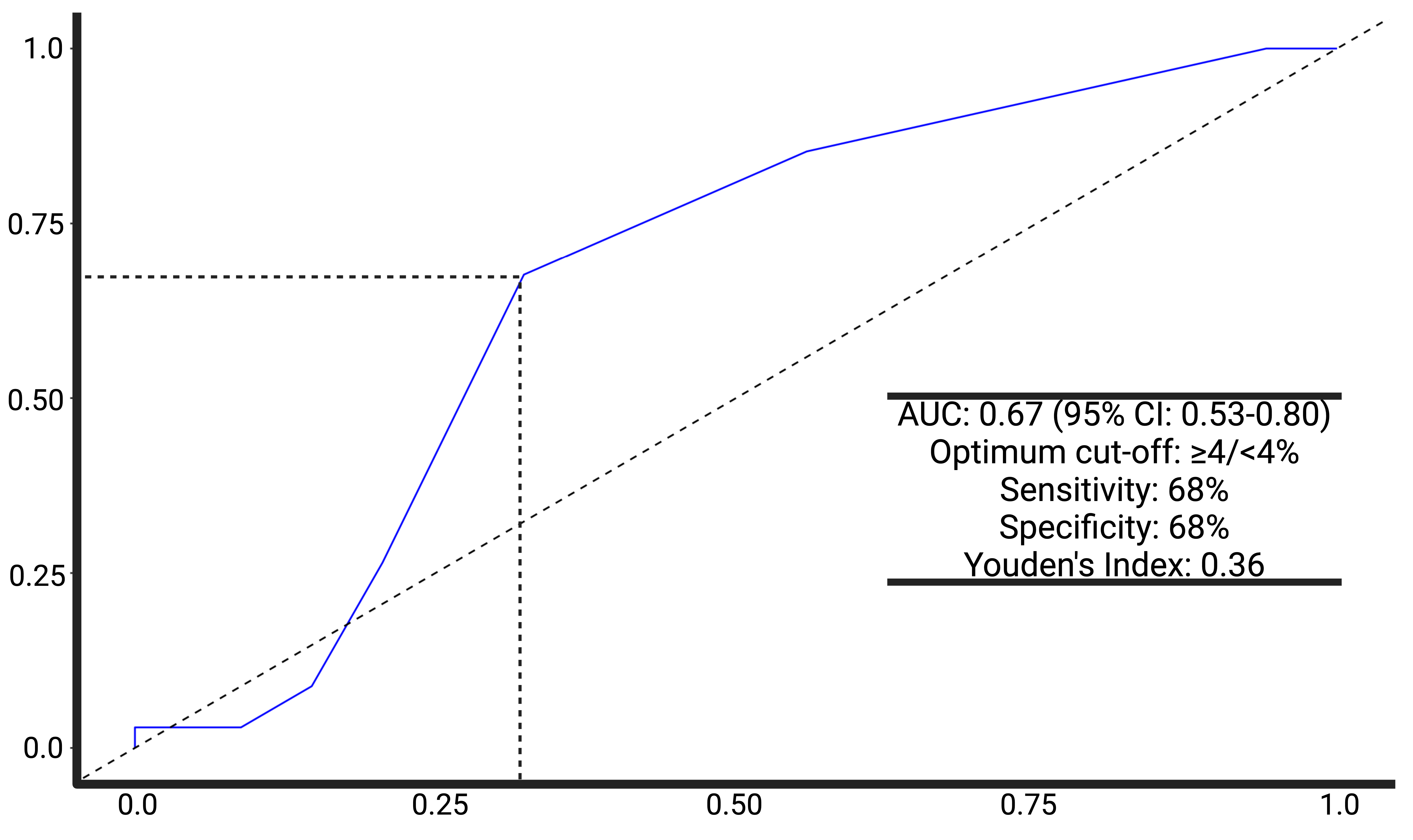

Supplement: Supplementary file 2 [file Image_1.tiff]
